# The landscape of DNA methylation in asthma: a data mining and validation

**DOI:** 10.1080/21655979.2021.1997088

**Published:** 2021-12-07

**Authors:** Hui Yang, Fei-yang Na, Li Guo, Xuan Liang, Rong-Fang Zhang

**Affiliations:** The Area B of International Medical Department, Gansu Provincial Maternity and Child-care Hospital, Lanzhou, Gansu, People’s Republic of China

**Keywords:** Asthma, DNA methylation, biomarker, HLA-DMB

## Abstract

Human asthma is caused by interactions between a range of genetic and environmental factors. However, the specific pathogenesis of asthma remains controversial. This study explored the contribution of DNA methylation to asthma using computer learning methods. Relevant datasets and information related to patients with asthma were collected from the Gene Expression Omnibus (GEO) database. A multivariate linear regression model was established. Differentially expressed genes and DNA methylation sites were identified. The results showed that the expression of 169 genes was significantly different between the two groups. Through differential analysis of methylation and differential analysis of gene expression, 44 differentially expressed genes that may be affected by DNA methylation modification were identified. The results of the multiple linear regression model showed that DNA methylation could explain 9.81% of the variation in gene expression. Gene ontology and Kyoto Encyclopedia of Genes and Genomes analyses showed that the differentially expressed genes, *HLA-DMB, IL4, HLA-DPB1*, and *CD40LG*, were related to the occurrence of asthma, and *HLA-DMB* expression was significantly reduced in allergic asthma. There was a positive correlation between cg04933135 and *HLA-DMB* expression, and cg04933135 was a differential site for DNA methylation. Using blood samples from asthma patients, we confirmed that *HLA-DMB* expression is down-regulated, which may be affected by abnormal DNA methylation. DNA methylation plays an important role in the development of asthma, and HLA-DMB which modified by abnormal DNA methylation can be regarded as a new biomarker of asthma.

## Introduction

Asthma is a common respiratory disease, with an incidence of 1–18% in different countries. The incidence and mortality of asthma are increasing annually worldwide [1,2]. The pathology of asthma includes chronic airway inflammation, airway hyperresponsiveness, and airway remodeling [2]. A variety of cells and inflammatory factors are involved in asthma, mostly due to repeated exposure to specific allergens, and the disease manifests as extensive reversible airflow limitation [3]. Human asthma is caused by the interaction of a range of genetic and environmental factors [4]. However, the specific pathogenesis of asthma remains controversial.

DNA methylation is one of the most characteristic, earliest discovered, and most important epigenetic modifications [5]. DNA methylation can change genetic expression without changing the underlying DNA sequence, producing changes in chromatin structure, DNA conformation, DNA stability, and interactions between DNA and protein, thereby controlling gene expression [6]. Breton et al. reported that PM2.5 exposure is associated with the methylation of CpG sites on the *NOS* gene [7]. Increased exposure to environmental air pollutants has been reported to significantly increase the methylation level of the *FOXP3* gene, affecting the normal function of Treg cells and increasing the incidence of asthma [8]. Fu et al. analyzed the whole blood of 60 children with mild asthma and 122 children with severe asthma and found that the hypermethylation of the 5′-UTR of the β-adrenergic receptor correlated with the severity of asthma [9]. Using genome-wide association studies (GWAS), several genetic mechanisms related to asthma have been discovered, and epigenetics has also shed new light on the pathogenesis of asthma.

Relevant data sets and information related to patients with asthma were collected from the Gene Expression Omnibus (GEO) database. A multivariate linear regression model was established, incorporating sex, age, batch effects, and the influence of methylation on gene expression, with the aim of explaining the contribution of various factors to asthma. Differentially expressed genes and DNA methylation sites were screened to explore the genetic mechanisms underlying asthma by correcting for the effects of sex, age, and peers on gene expression. In the current study, we analyzed the contribution of DNA methylation to asthma through bioinformatics methods and validation in patient samples to help us understand the pathogenesis of asthma.

## Materials and methods

### DNA methylation and mRNA expression microarray data

The GSE104472 Series Matrix File data file was downloaded from the GEO database of the National Center for Biotechnology Information (NCBI) (https://www.ncbi.nlm.nih.gov/geo/). GSE104472 contains data from the nasal epithelial tissue of 12 patients with allergic asthma and 12 healthy controls, including corresponding mRNA expression data collected using an Agilent-072363 SurePrint G3 Human GE v3 8x60K Microarray, and a DNA methylation chip (Illumina HumanMethylation450 BeadChip) [10]. The DNA methylation chip contained the M values of 485,577 methylation probes. The M value was calculated as the logarithm of the ratio of the methylated signal intensity to the intensity of the unmethylated signal [11]. A larger M value indicates a higher degree of methylation. The mRNA expression chip contained the expression values of 58,341 probes. The expression levels of the genes were normalized and subjected to log_2_ transformation.

### Methylation differential analysis

When performing methylation differential analysis, methylation probes of both a methylation site and single nucleotide polymorphism (SNP) site were excluded. T-tests were used to compare differences in methylation between patients with allergic asthma and healthy controls. The Benjamini and Hochberg method was used to correct the *p*-value. A corrected *p*-value <0.05, and an average difference of M-value >0.3 was used as the cutoff to screen the methylation differential probes. The circlize R package was used to draw a circos map of the distribution of differentially methylated sites, including hypomethylation and hypermethylation sites, in each 10 Mb window on each chromosome [12]. A ratio of the number of hypomethylation sites to the number of hypermethylation sites greater than 1.5 indicates that a hypomethylated functional domain exists on the chromosome. A ratio of the number of hypermethylation sites to the number of hypomethylation sites greater than 1.5 suggests that a hypermethylated functional domain exists on the chromosome.

### Gene expression differential analysis

T-tests were used to analyze the differences in expression between patients with allergic asthma and healthy controls. Genes with *p* < 0.05, and |log_2_FC| > 2 were selected as differentially expressed genes.

### Correlation between methylation sites and gene expression

A multivariate linear regression model was established to explore the correlation between methylation and gene expression. Covariates, including sex, age, and batch effects, were added for model correction. The methylation sites of the promoter region and adjacent gene, at a distance of 1 Mb, were selected for analysis. After correcting for the effects of sex, age, and peers on gene expression, results with *p* < 0.05, were selected for subsequent analysis [13].

### Gene enrichment analysis

ClusterProfiler was used for gene functional annotation analysis and the Kyoto Encyclopedia of Genes and Genomes (KEGG) pathway cluster analysis of genes related to differential methylation sites [14,15]. The Benjamini & Hochberg method was used for *p*-value correction. A corrected *p*-value <0.05, minimum number of genes per pathway of 10, and maximum number of genes of 500 were taken as the standards.

### Sample collection

In the current study, we collected peripheral blood samples from 30 asthma patients and 30 healthy volunteers. Add anticoagulant to the above blood sample for anticoagulation, and then store at −80°C. Participants provided written consent, and the study was approved by the Ethics Committee of Gansu Provincial Maternal and Child Health Hospital.

### RNA extraction and RT-qPCR

Total RNA was extracted from peripheral blood using with Trizol reagent (Invitrogen, Grand Island, NY, USA) according to manufacturer’s protocols the product instructions. Reverse transcription of into cDNA was synthesized using with PrimeScript RT Reagent Kit (Takara, Shiga, Japan) according to the product specifications manufacturer’s protocols. RT-qPCR was performed with using SYBR Green PCR Master Mix (Takara) in Applied Biosystems ABI 7500 (Applied Biosystems, Waltham, MA, USA). The primers for HLA-DMB mRNA are as follows: F: 5ʹ-ACCTGTCTGTTGGATGATGCT-3ʹ; R: 5ʹ-CGCAAGGGGCCATCTTATTCT-3ʹ.

### Methylation specific (MSP)-PCR

Peripheral blood genomic DNA was extracted strictly according to the genomic DNA extraction kit instructions (Tiangen Biotech, Beijing, China). According to the standard protocol, DNA Methylation Gold Bisulfite Conversion Kit (Zymo Research, Irvine, USA) was used to perform bisulfite conversion of genomic DNA. Primers specific to methylated or un-methylated promoter regions were designed using online tools (Table 1). The PCR reaction system was as follows: 10× PCR Buffer 5.0 μl, 5 mM dNTPs 5.0 μl, Primer F 2.0 μl, Primer R 2.0 μl, methylation recovery products 1.5 μl, 5 U/μl Taq HS 0.2 μl, ddH2O 34.3 μl. The cycling protocol was as follows: pre-denaturation 95°C for 5 min followed by 40 cycles of denaturation 95°C for 30s, annealing 55°Cfor 30s, extension 72°C for 30s, terminal extension 72°C for 5 min, cooling 16°C for 2 min.

**Table 1.** Primer for MSP-PCR

Name	Forward Primer (5ʹ-3ʹ)	Reverse Primer (5ʹ-3ʹ)
HLA-DMB (M)	AAATTTGTTTTTTTAAGATATATACGT	CAACAATATATAAACCTTCCGTT
HLA-DMB(UM)	TAAATTAAAATTATAATGAGATATTGT	ACCAACAATATATAAACCTTCCATT

M: methylation; UM: un-methylation.

### Statistical analysis

Fisher’s exact test was used to compare the methylation status of the HLA-DMB gene in blood samples from asthma patients and healthy volunteers. The unpaired t test was used to compare the expression level of HLA-DMB mRNA in blood samples of asthmatic patients and healthy volunteers. Use GraphPad Prism 7.0 software (GraphPad, California, USA) for statistical analysis.

## Results

DNA methylation chip and mRNA expression chip data were downloaded from the GEO database. Affymetrix genome chip platforms GPL13534 and GPL21185 were used to annotate methylation probes and mRNA expression chips, respectively. Probes with significant correlation between methylation differences and gene expression were selected as candidate methylation sites to study the correlation between DNA methylation and gene expression, and the function of genes in allergic asthma.

### Distribution of differentially methylated sites

**Figure 1.**
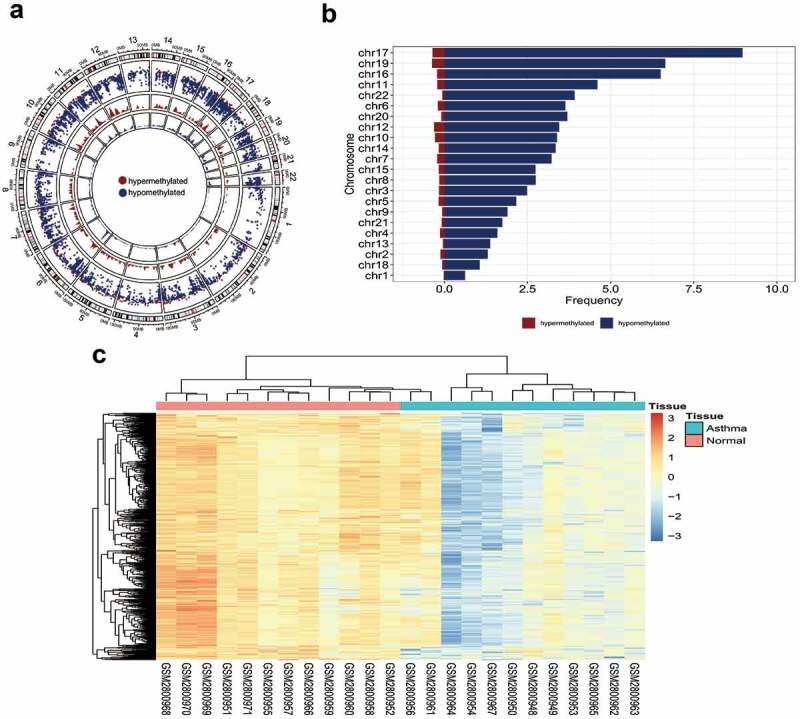
**DNA methylation in asthma**. (a) The distribution of differential methylation sites on chromosomes; (b) Frequency of hypermethylation and hypomethylation on each chromosome; (c) Clustering heat map of methylation difference sites

First, we analyzed the distribution of differential methylation sites and used the downloaded data to get a preliminary understanding of the status of DNA methylation between asthma patients and healthy people. A total of 351,596 probes were used for differential methylation analysis after screening the methylation probes. Among them, 8,119 methylation sites showed significant statistical differences (FDR<0.05, |M-value|>0.3) (**Figure S1A**). |M-value| was defined as the difference between the mean value of the asthma patient tissue and the mean M-value of the healthy tissue. Compared with healthy tissues, 94.65% (8,119) of sites in patients with asthma were hypomethylated, and 5.35% (459) sites were hypermethylated. Thus, a hypomethylation was more obvious than hypermethylation in tissue from patients with asthma at the genome-wide level. The methylation modifications of each chromosome are shown in Figure 1a. The inner circle used 10 M as a window to demonstrate the depth of hypermethylation and hypomethylation.

We observed that chromosomes 17, 19, and 16 had the largest number of differentially methylated sites, while chromosome 1 had the least number of differentially methylated sites (Figure 1b). The highest degree of methylation was 8.97/Mb, whereas the lowest degree of methylation was 0.02/Mb. By comparing the ratio of hypomethylation to hypermethylation on each chromosome, we concluded that each chromosome had a hypomethylated pattern, with chromosome 22 having the highest ratio (Table 2), which further demonstrated that hypomethylation was more widespread than hypermethylation in tissue from patients with asthma. The analysis revealed no differential methylation sites on sex chromosomes. Consistent with the results of previous analyses, there was no significant difference in methylation sites between patients with asthma and healthy controls [10].

**Table 2.** Distribution of methylation on each chromosome

Chr	Length	Hyper/Mb	Hypo/Mb	CpG/Mb	Hyper/Hypo ratio
chr1	249.25	0.02	0.62	0.64	31.00
chr2	243.20	0.12	1.30	1.42	10.93
chr3	198.02	0.16	2.49	2.65	15.41
chr4	191.15	0.13	1.60	1.73	12.20
chr5	180.92	0.17	2.16	2.33	12.61
chr6	171.12	0.20	3.64	3.84	18.32
chr7	159.14	0.22	3.22	3.44	14.66
chr8	146.36	0.15	2.75	2.90	18.27
chr9	141.21	0.06	1.90	1.96	29.78
chr10	135.53	0.28	3.39	3.67	12.08
chr11	135.01	0.21	4.61	4.82	21.45
chr12	133.85	0.31	3.45	3.76	11.27
chr13	115.17	0.04	1.38	1.42	31.80
chr14	107.35	0.17	3.34	3.51	19.94
chr15	102.53	0.17	2.74	2.91	16.53
chr16	90.35	0.22	6.51	6.73	29.40
chr17	81.20	0.36	8.97	9.32	25.10
chr18	78.08	0.06	1.06	1.13	16.60
chr19	59.13	0.37	6.65	7.02	17.86
chr20	63.03	0.10	3.70	3.79	38.83
chr21	48.13	0.08	1.75	1.83	21.00
chr22	51.30	0.06	3.92	3.98	67.00

Of the differentially methylated sites, 48.44% were in the promoter region (promoter, 5′-UTR, and first exon), and 51.56% were in the remaining positions (**Figure S1B**). Nearly half of the differentially methylated sites were located near the transcription initiation site, indicating that these sites may regulate gene transcription activity. Rank sum tests were used to compare methylation levels in different regions. Although the methylation levels of the intron and downstream regions were not significantly different, the methylation of promoter regions generally showed a low methylation pattern. Therefore, hypomethylation sites in the promoter region were used for subsequent analyses. In addition, the top 500 differentially methylated sites were used for clustering, which could distinguish asthma tissues from normal tissues (Figure 1c).

### Differential analysis of gene expression

**Figure 2.**
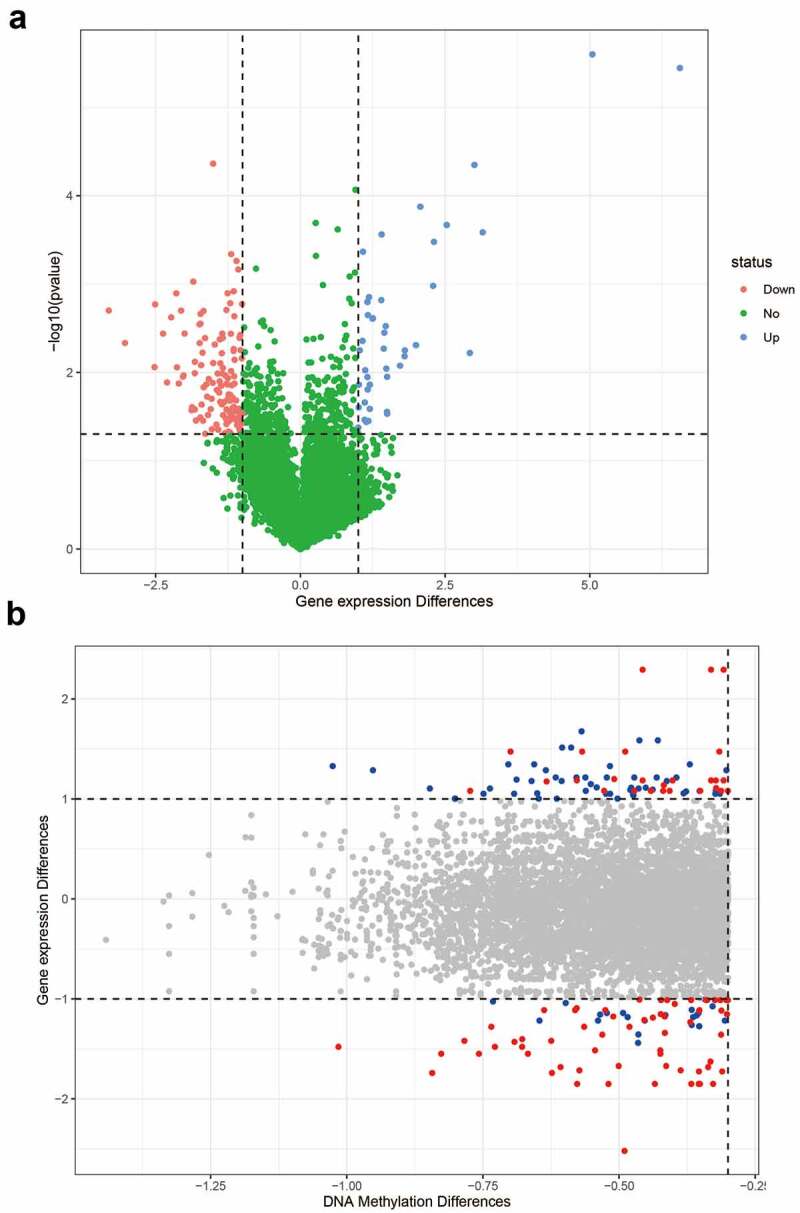
**Differential genes in asthma**. (a) Volcano map of differential genes; (b) The relationship between DNA methylation differences and expression of differential genes. The blue dots indicate that the fold change of gene expression was greater than 2, and the average difference of methylation sites was greater than 0.3. The red dots indicate the sites with statistical differences

Next, we analyzed which differential genes may be modified by abnormal DNA methylation in asthma. Based on the correspondence between the chip probes and genes, 21,457 probes were matched to gene names. Since multiple probes corresponded to the same gene, the average expression of different probes was used for the expression of a specific gene. A total of 16,903 genes were identified for differential gene expression analysis. The expression of 169 genes was significantly different, with a fold change >2 (*p* < 0.05) (Figure 2a), and the expression of 507 genes was significantly different, with a fold change >1 (*p* < 0.05). According to the differential analysis of methylation and gene expression, 44 differentially expressed genes may have been affected by methylation modifications (Figure 2b). Hypomethylated sites in the promoter region were selected for subsequent analysis to further explore the correlation between DNA methylation and gene expression.

### Correlation analysis between methylation sites and gene expression

**Figure 3.**
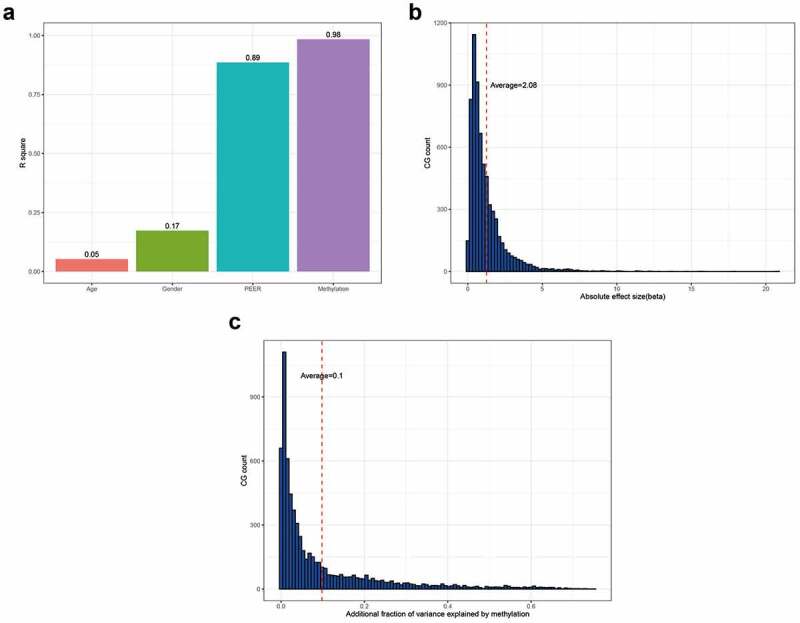
**Analysis of the correlation between DNA methylation sites and gene expression**. (a) Cumulative ratio of gene expression variation; (b) Beta size histogram distribution; (c) Interpreted histogram distribution of gene expression variation

The number of peers was set at five, followed by the addition of covariates, such as age and sex, for correction. There were 6,642 significant CG-gene relationship pairs (182 methylation sites and 155 genes). Specifically, 2,068 methylation sites negatively correlated with the expression of 1,568 genes, and 1,898 methylation sites positively correlated with the expression of 1,293 genes. The addition of age to the regression model could explain an average of 5.24% of the gene expression variation. The addition of sex to the regression model could explain an average of 17.32% of gene expression variation. The addition of peers explained 88.62% of the variation in gene expression. The addition of methylation to the model could explain 98.43% of the variation in gene expression. After correction by sex, age, and peers, methylation explained 9.81% of the variation in gene expression (Figure 3a). The beta size ranged from 0.02 to 20.82, with an average effect size of 2.08 (Figure 3b). DNA methylation could explain the variation in gene expression (range: 0.016–75.14%) (Figure 3c).

### Gene ontology annotation and KEGG analysis

**Figure 4.**
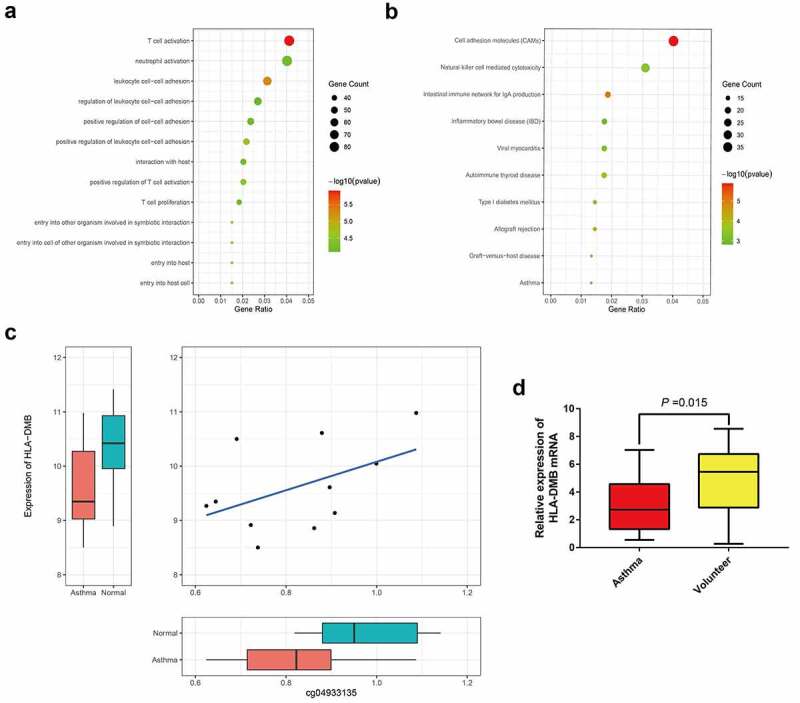
**GO annotation and KEGG analysis**. (a) Gene function annotation analysis; (b) KEGG pathway analysis; (c) Expression correlation between cg04933135 and *HLA-DMB*; (d) The expression level of *HLA-DMB mRNA* was detected in the blood samples of asthma patients and healthy volunteers by RT-qPCR

Gene functional annotation analysis (Figure 4a) and KEGG pathway analysis (Figure 4b) were performed on 1,967 genes. Gene ontology (GO) functional annotation analysis revealed that 87 genes were involved in T-cell activation. KEGG pathway analysis revealed that 13 genes were involved in the asthma pathway. The genes identified by both analyses included *HLA-DMB, IL4, HLA-DPB1*, and *CD40LG. HLA-DMB* expression was significantly lower in patients with allergic asthma (*p* = 0.0275, log_2_FC = −1.12), cg04933135 correlated with *HLA-DMB* expression (*p* = 0.034), and cg04933135 was the site of differential methylation (corrected *p* = 0.041). The above findings show that the abnormal methylation of cg04933135 in allergic asthma tissue led to differential expression of the *HLA-DMB* gene. The expression of *HLA-DMB* was lower in the allergic asthma tissue, changing the maturation of T lymphocytes, thereby leading to the occurrence of asthma.

### Expression of HLA-DMB in asthma and its DNA methylation status

In order to verify the results of bioinformatics analysis, we collected blood samples from asthma patients and healthy volunteers to detect the expression level of *HLA-DMB mRNA* and its DNA methylation status. RT-qPCR results showed that the expression level of HLA-DMB mRNA in the blood samples of asthma patients was significantly lower than that of healthy volunteers, indicating HLA-DMB is closely related to the occurrence of asthma (Figure 4d). On the other hand, we analyzed the status of *HLA-DMB* methylation in the blood samples of asthma patients and healthy volunteers by MSP-PCR. The results showed that methylation of *HLA-DMB* was detected in 90.0% (27/30) of patients with asthma, and methylation was detected in 3.3% (1/30) of healthy volunteers. The methylation status of *HLA-DMB gene* in the blood samples of asthma patients and healthy controls was significantly different (*P* < 0.001). In addition, the un-methylation of HLA-DMB accounted for 10.0% and 96.7% of asthma patients (3/30) and healthy volunteers (29/30), respectively. Therefore, abnormal DNA methylation may be an important reason for the low expression of HLA-DMB in asthma.

## Discussion

An in-depth investigation of molecular genetics has revealed that epigenetic modifications, such as DNA methylation, alternative splicing, and non-coding RNA regulation, can alter biological characteristics. For example, Wang et al. summarized the research progress of the role of miRNAs, lncRNAs, circRNAs and ceRNA regulatory networks in asthma through bioinformatics methods [16]. DNA methylation is the most important mechanism of modification, primarily affecting gene expression and cell differentiation and development [17]. Asthma is a chronic inflammatory disease of the respiratory tract, which is closely associated with an imbalance of Th1/Th2 cells and Th2/Treg cells [18,19]. Epigenetic research on asthma, especially methylation, is a hot topic in the field of asthma pathogenesis [20,21].

In this study, bioinformatics analysis of DNA methylation sequence data from patients with asthma revealed that the expression of a total of 507 genes was significantly different (*p* < 0.05, fold change >1). Differential analysis of methylation and gene expression demonstrated that 44 differentially expressed genes may be affected by methylation modifications. There were 6,642 significant CG-gene relationship pairs (182 methylation sites and 155 genes). Specifically, 2,068 methylation sites negatively correlated with the expression of 1,568 genes, and 1,898 methylation sites positively correlated with the expression of 1,293 genes. Further analysis of the regression model revealed that the addition of age and sex could explain 17.32% of gene expression variation. The addition of peers could explain up to 88.62% of the variation in gene expression. The addition of methylation could explain 98.43% of the variation in gene expression. After correction for sex, age, and peers, methylation explained 9.81% of the variation in gene expression. Therefore, we believe that DNA methylation plays a vital role in the pathogenesis of asthma. Nadeau et al. found that Treg cells are important immune response inhibitors in the pathogenesis of asthma [8]. Treg cell injury is associated with *FOXP3* gene methylation, and FOXP3 is a key transcription factor for Treg cell activity [22]. Studies have also reported that demethylation of the *IL4* gene promoter region is enhanced by the stimulation of allergens, which helps the differentiation of naive T lymphocytes into Th2 cells. The *HLA-DMB* gene has been reported to be essential for the formation of MHC II/peptide complexes in antigen-presenting cells [23]. Subsequent studies by Pezeshki et al. also found that the forced expression of HLA-DM on the surface of dendritic cells increased the load of synthetic peptides of MHC II molecules and Treg cell responses [24].

In this study, GO functional annotation analysis further revealed that 87 genes were involved in T cell activation. The intersecting genes were *HLA-DMB, IL4, HLA-DPB1*, and *CD40LG. HLA-DMB* expression was significantly decreased in allergic asthma, and the differentially methylated site cg04933135 positively correlated with *HLA-DMB* expression. This observation showed that the abnormal methylation of cg04933135 in allergic asthma tissue could lead to differential expression of the *HLA-DMB* gene. The decreased expression of *HLA-DMB* in allergic asthma tissue affected the maturation of T lymphocytes, thereby causing asthma. However, no study has reported that abnormal methylation of the *HLA-DMB* gene promotes the maturation of T lymphocytes to trigger asthma. Further analysis of *HLA-DMB* gene methylation, its differentially methylated site cg04933135, and T lymphocyte levels with respect to the occurrence of asthma. Finally, we collected blood samples from asthma patients and healthy people, and detected the expression level of *HLA-DMB mRNA* and the methylation status of *HLA-DMB*. As expected, the expression level of *HLA-DMB mRNA* in blood samples of asthma patients is lower than that of healthy people. Our further analysis revealed that *HLA-DMB* has abnormal DNA methylation modification, which may be one of the main reasons for the low expression of HLA-DMB in asthma.

## Conclusions

In summary, DNA methylation plays an important role in the development of asthma, and HLA-DMB which modified by abnormal DNA methylation can be regarded as a new biomarker of asthma. Our study may provide new theoretical bases for elucidating the pathogenesis of asthma.

## Supplementary Material

Supplemental Material

## Data Availability

The data used to support the findings of this study are available from the corresponding author (gsfy_zrf@163.com) upon request.

## Highlights

1. DNA methylation contributes to the onset of asthma.
2. HLA-DMB expression is significantly reduce in allergic asthma.
3. DNA methylation of HLA-DMB is closely related to asthma.
