# Supplementary figures and images for: The landscape of DNA methylation in asthma: a data mining and validation
Source: Bioengineered. 2021 Dec 7;12(2):10063–72. doi: 10.1080/21655979.2021.1997088 (PMC8809922; doi:10.1080/21655979.2021.1997088)

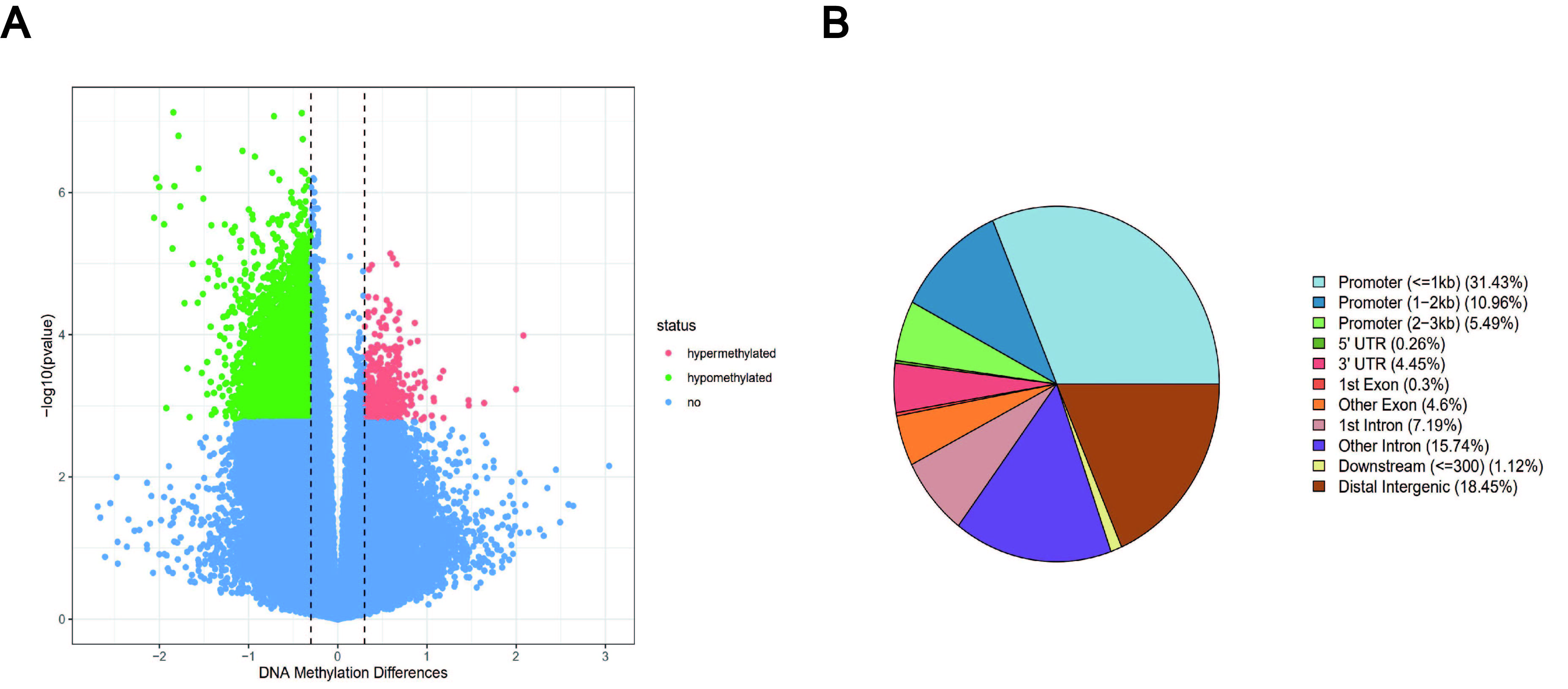

Supplement: Supplemental Material [file KBIE_A_1997088_SM8527.jpg]
